# Add-on effect of the Guizhi Fuling formula for management of reduced fertility potential in women with polycystic ovary syndrome: A systematic review and meta-analysis of randomized controlled trials

**DOI:** 10.3389/fendo.2022.995106

**Published:** 2023-04-18

**Authors:** Anna Rong, Na Ta, Lihua E., Wenbin Meng

**Affiliations:** ^1^ Department of Obstetrics and Gynecology, The Affiliated Hospital of Inner Mongolia Medical University, Hohhot, China; ^2^ Department of Stomatology, The Affiliated Hospital of Inner Mongolia Medical University, Hohhot, China

**Keywords:** Guizhi Fuling capsule/pill, insulin resistance, meta-analysis, polycystic ovary syndrome, pregnancy, sex hormone

## Abstract

**Background:**

Guizhi Fuling (GZFL) pill, a traditional Chinese herbal formula including *Semen Persicae*, *Ramulus Cinnamomi*, *Poria*, *Radix Paeoniae Alba*, and *Cortex Moutan*, has been widely applied in the management of gynecological diseases.

**Objective:**

To evaluate the add-on effect of the GZFL formula for treating reduced fertility potential in women with polycystic ovary syndrome (PCOS) by conducting a systematic review and meta-analysis.

**Methods:**

Two reviewers independently searched the PubMed, Embase, Cochrane Library, Wanfang, SinoMed, and CKNI databases until 09/11/2022. Eligible studies were randomized controlled trials (RCTs) of the GZFL formula plus Western medicine versus the Western medicine for treating PCOS. The primary endpoint was the ovulation, pregnancy, and miscarriage rate. The secondary endpoints included the serum follicle-stimulating hormone (FSH), total testosterone, luteinizing hormone (LH), estradiol, and homeostasis model assessment insulin resistance (HOMA-IR).

**Results:**

There were 16 RCTs with 1,385 patients identified. The GZFL formula plus Western medicine significantly improved the ovulation rate (risk ratios [RR] 1.24; 95% confidence intervals [CI] 1.15–1.34) and pregnancy rate (RR 1.53; 95% CI 1.38 to 1.69) than the Western medicine alone. Adjuvant treatment with the GZFL formula also significantly decreased the serum FSH (mean difference [MD] -0.48 U/l; 95% CI -0.80 to -0.15), total testosterone (standard mean difference [SMD] -1.07; 95% CI -1.71 to -0.44), LH level (MD -2.19 U/l; 95% CI -3.04 to -1.34), and HOMA-IR (MD -0.47; 95% CI -0.60 to -0.34). However, there was no significant difference in the miscarriage rate (RR 0.89; 95% CI 0.36–2.20) and serum estradiol level (SMD 0.34; 95% CI -0.25 to 0.94) between two groups.

**Conclusions:**

The GZFL formula as adjuvant therapy can improve the ovulation and pregnancy rates in women with PCOS. Its beneficial effects may correlate with reducing FSH, total testosterone, and LH and ameliorating insulin resistance. However, more well-designed RCTs with larger samples and multicenter trials are required to confirm the current findings due to uncertainty of the evidence.

**Systematic review registration:**

PROSPERO identifier, CRD42022354530.

## Introduction

Polycystic ovary syndrome (PCOS) is a hormonal disorder common among reproductive-age women. The pooled mean prevalence of PCOS was 21.27% using different diagnostic criteria (1). Women with PCOS are more likely to develop certain long-term health sequelae including type 2 diabetes, metabolic syndrome, and endometrial cancer (2). Apart from hormonal imbalance and metabolic problems, fertility reduced in ovulatory women with PCOS is also a big concern (3). Alterations in oocyte competence are considered potential causative factors for subfertility in women with PCOS (4). Management of fertility reduced in ovulatory women with PCOS include lifestyle changes, pharmacological ovulation induction, reproductive technologies, or laparoscopic ovarian drilling (5). However, achievement of successful fertility in women with PCOS remains a major concern (6).

Traditional Chinese medicine (TCM) has been used to treat gynecological diseases including PCOS. The Guizhi Fuling (GZFL) formula was firstly described in Jingui Yaolue of the Han dynasty. This prescription includes *Semen Persicae*, *Ramulus Cinnamomi*, *Poria*, *Radix Paeoniae Alba*, and *Cortex Moutan*. This classical formula exhibits the effects of activating blood and dissolving blood stasis according to TCM theory. The combination of the GZFL capsule/pill has been widely applied for treatment of PCOS (7, 8). A previous meta-analysis published in Chinese (9) has concluded that the GZFL formula combined with Western medicine was superior to the Western medicine in improving the ovulation and pregnancy rate in women with PCOS. However, the impact of the GZFL formula on sex hormone level and insulin resistance was not well-characterized in this meta-analysis.

No previous meta-analysis published in English literature has specially focused on the add-on effect of the GZFL formula for management of reduced fertility potential in women with PCOS. To address this knowledge gap, we conducted a systematic review and meta-analysis of randomized controlled trials (RCTs) to evaluate the add-on effect of the DZFL formula for treatment of infertility associated with PCOS.

## Methods

### Literature search

The current study was performed and reported based on the guidelines of Preferred Reporting Items for Systematic Reviews and Meta-Analyses (10). Our study was registered in the PROSPERO database (CRD42022354530). Two reviewers independently searched PubMed, Embase, Cochrane Library, Wanfang, VIP, SinoMed, and China National Knowledge Infrastructure databases until 09/12/2022. Keywords for the literature search included the following (**Supplemental Text S1**): “Gyejibokryeong-Hwan” OR “Guizhi Fuling” OR “Gui zhi Fu ling” AND “polycystic ovary syndrome” OR “polycystic ovarian syndrome” OR “PCOS” AND “randomized controlled trial” OR “random.” Reference lists of retrieved studies and reviews were also manually searched to identify any additional eligible studies.

### Study selection

Studies satisfying the following criteria were included: 1) patients with a clinical diagnosis of PCOS; 2) study design: RCTs; 3) GZFL formula regardless of capsule, pill, or decoction plus Western medicine versus the same Western medicine alone as intervention; and 4) the primary endpoint was the ovulation rate, pregnancy rate, and miscarriage rate. The secondary endpoints included the serum follicle-stimulating hormone (FSH), total testosterone, luteinizing hormone (LH), estradiol level, and homeostasis model assessment insulin resistance (HOMA-IR). Exclusion criteria included the following: 1) modified GZFL formula as intervention; 2) GZFL formula combined with any complementary therapy as intervention; 3); any different treatment except for the GZFL formula between two groups; 4) patients with Cushing’s syndrome or congenital adrenal hyperplasia, and 5) duplicate publication or suspected plagiarism.

### Data extraction and quality assessment

The following data were collected by two independent reviewers from the selected trials: name of the first author, publication time, number of patients, mean age or age range, type/dosage of GZFL, detailed Western therapy, duration of intervention, duration of follow-up, outcome measures, and quality assessment information. The Cochrane Collaboration risk-of-bias tool was applied to evaluate the methodological quality of eligible trials, which assesses randomization generation, allocation concealment, blinding of participants and personnel, blinding of outcome assessors, incomplete outcome data, selective outcome reporting, and whether to enroll patients according to TCM syndrome. Any disagreement between the two reviewers was settled by consensus or asked for the third reviewer.

### Data analysis

All data were analyzed using Review Manager version 5.1 and STATA 12.0 (STATA Corp LP, College Station, TX, USA). The effect sizes were summarized by pooling weight mean difference (WMD) or standard mean difference (SMD) with a 95% confidence interval (CI) for the continuous outcome data. For the binary outcome data, we pooled the risk ratios (RR) with 95% CI for the GZFL formula plus Western medicine versus the Western medicine alone. Heterogeneity across trials was examined using the I^2^ statistic and Cochrane Q test. A random-effect model was selected when there was significant heterogeneity (I^2^ statistic ≥50% and/or *p*-value <0.1 of the Cochrane Q test); otherwise, we selected a fixed-effect model. To investigate the robustness of the pooling effect size, we conducted a leave-out one trial sensitivity analysis. Subgroup analysis was conducted according to the types of Western medicine, course of treatment, and form of prepared GZFL formula. Begg’s test (11) and Egger’s test (12) were used to assess publication bias. In the case of significant publication bias, the trim-and-fill analyses were used to correct the pooling effect size. The GRADE method was used to summarize the certainty of evidence.

## Results

### Search results and study characteristics

**Figure 1:**
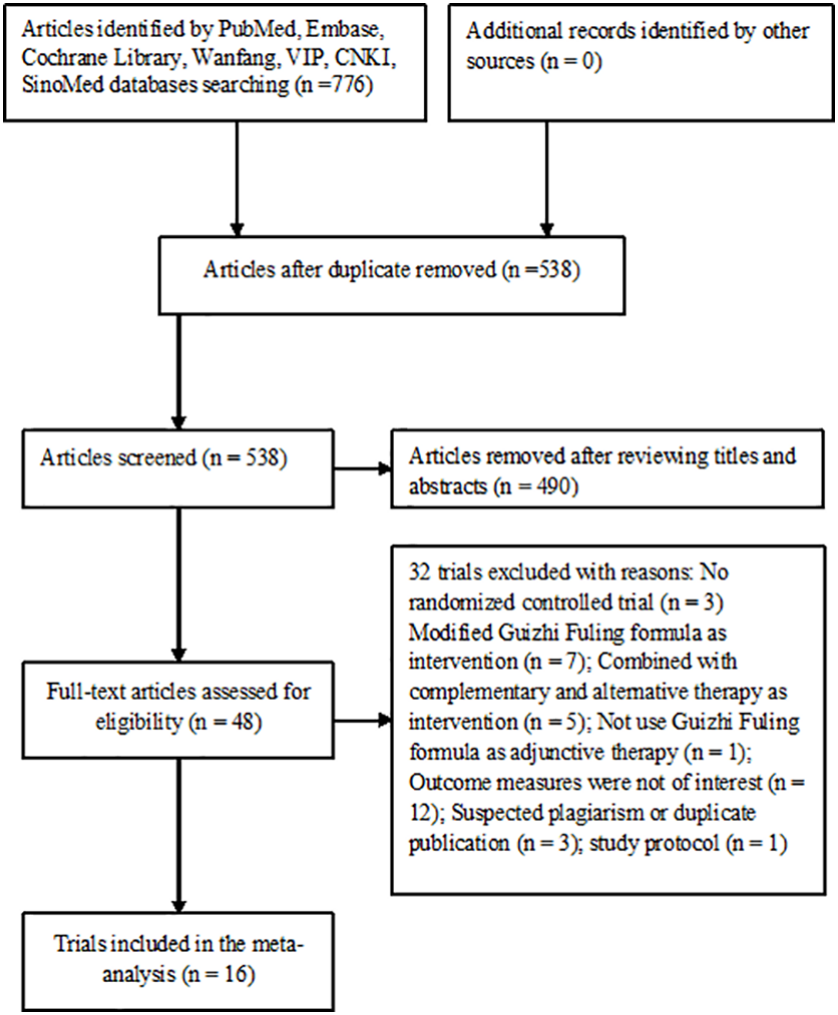
Flowchart showing the trial selection process.

The literature search identified 776 articles, of which 538 articles were left after exclusion of duplicates. After reviewing the titles and abstracts, 490 articles were excluded and then 48 articles were left for full-text evaluation. Finally, 16 trials (13–28) were included in the meta-analysis after applying the predefined criteria for inclusion and exclusion. A flowchart of the study selection is shown in **Figure 1**.

The main features of the included trials are shown in **Table 1**. These eligible trials were published between 2008 and 2022. All the included trials were conducted in China and published in Chinese. A total of 1,385 women with PCOS were identified. The duration of treatment varied from three to six menstrual cycles. For each course of treatment, the GZFL formula was administered at a dosage of 2.79 to 12 mg daily except for the menstrual period. Clomiphene was administered at a dosage of 50 to 100 mg daily from the 5th to 10th days of menstruation. Ethinylestradiol/cyproterone acetate (ECA) 2 mg/0.035 mg daily was administered from the 5th day of menstruation for 21 consecutive days. **Supplemental Figure S1** and **Figure S2** summarize the risk of bias of included trials. According to the Cochrane Collaboration risk-of-bias tool, most of the trials were classified as suboptimal methodological quality with an unclear risk of bias. Only two trials (15, 28) enrolled the patients based on the TCM syndrome differentiation.

**Table 1:** Main features of clinical trials included in the meta-analysis.

Author/year	Sample size	Age (years)	Main interventions		Course of treatment	Follow-up duration	Outcome measures
			Outcome measures	Outcome measures			
Zhao HB 2008 (13)	GZFL:34 Con: 34	28-34	GZFL capsule 2.79 g/day + clomiphene citrate 50 mg/day × 5	Clomiphene citrate 50 mg/day × 5	3 months		Pregnancy, ovulation, LH, FSH, TT, HOMA-IR
Shi SQ 2010 (14)	GZFL:24 Con: 17	Not reported	GZFL capsule 2.79 g/day + clomiphene citrate 50 mg/day × 5	Clomiphene citrate 50 mg/day × 5	3 months		Pregnancy, ovulation, TT
Ye HJ 2012 (15)	GZFL:30 Con: 30	GZFL: 27.7 ± 3.6 Con: 27.8 ± 3.8	GZFL capsule 2.79 g/day + ECA 2 mg/0.035 mg/day + clomiphene citrate 50 mg/day × 5	ECA 2 mg/0.035 mg/day + clomiphene citrate 50 mg/day × 5	3 menstrual cycles		Pregnancy, ovulation, LH, TT
Shao JY 2016 (16)	GZFL:44 Con:44	GZFL: 26.1 ± 5.16 Con: 25.3 ± 4.54	GZFL capsule 2.79 g/day + clomiphene citrate 50 mg/day × 5	Clomiphene citrate 50 mg/day × 5	3 months		Pregnancy, ovulation, LH, FSH, TT, Estradiol
Zhang LY 2016 (17)	GZFL:55 Con: 55	GZFL: 30.8 ± 6.9 Con: 29.8 ± 6.6	GZFL pill 12 g/day + ECA 2 mg/0.035 mg/day + metformin 1.5 g/day	ECA 2 mg/0.035 mg/day + metformin 1.5 g/day	3 menstrual cycles		Pregnancy, ovulation, miscarriage, LH, FSH, TT, Estradiol, HOMA-IR
He WJ 2017 (18)	GZFL:45 Con: 32	GZFL: 27.49 ± 6.11 Con: 27.85 ± 6.18	GZFL pill 12 g/day + ECA 2 mg/0.035 mg/day	ECA 2 mg/0.035 mg/day	3 menstrual cycles		Pregnancy, ovulation, LH, TT
Tian Y 2017 (19)	GZFL:54 Con: 53	GZFL: 29.8 ± 4.5 Con: 29.3 ± 4.1	GZFL capsule 2.79 g/day + ECA 2 mg/0.035 mg/day + metformin 1.0 g/day	ECA 2 mg/0.035 mg/day + metformin 1.0 g/d	3 menstrual cycles		Pregnancy, ovulation, LH, FSH, TT, Estradiol, HOMA-IR
Wang ZY 2017 (20)	GZFL:52 Con: 52	GZFL: 28.02 ± 3.81 Con: 27.21 ± 4.17	GZFL capsule 2.79 g/day + ECA 2 mg/0.035 mg/day	ECA 2 mg/0.035 mg/day	3 months	3 months	Pregnancy, ovulation, LH, FSH, Estradiol
Cui YJ 2018 (21)	GZFL:28 Con: 28	GZFL: 28.0 ± 3.8 Con: 28.3 ± 2.5	GZFL tablet 2.88 g/day + ECA 2 mg/0.035 mg/day	ECA 2 mg/0.035 mg/day	3 menstrual cycles	6 months	Pregnancy, ovulation, TT
Luo J 2018 (22)	GZFL:54 Con: 54	GZFL: 30.23 ± 2.19 Con: 29.65 ± 2.47	GZFL pill 12 g/day + ECA 2 mg/0.035 mg/day + clomiphene citrate 50 mg/day × 5	ECA 2 mg/0.035 mg/day + clomiphene citrate 50 mg/day × 5	3 menstrual cycles	12 months	Pregnancy, miscarriage, LH, FSH, TT, estradiol
Wu ZW 2018 (23)	GZFL:40 Con:40	GZFL: 28.34 ± 3.59 Con: 28.16 ± 3.64	GZFL capsule 2.79 g/day + ECA 2 mg/0.035 mg/day	ECA 2 mg/0.035 mg/day	6 menstrual cycles	12 months	Pregnancy
Cui Y 2019 (27)	GZFL:79 Con:78	GZFL: 28.97 ± 2.92 Con: 29.03 ± 3.08	GZFL pill 6–12 g/day + pioglitazone 15 mg/day	Pioglitazone 15 mg/day	3 menstrual cycles	6 months	Pregnancy, ovulation
Zhang Y 2019 (24)	GZFL:28 Con:28	GZFL: 25-40 Con: 24-38	GZFL capsule 2.79 g/day + ECA 2 mg/0.035 mg/day + metformin 1.0 g/day	ECA 2 mg/0.035 mg/day + metformin 1.0 g/day	3 menstrual cycles		Pregnancy, ovulation
Zhao XH 2019 (25)	GZFL:44 Con: 44	GZFL: 31.7 ± 3.6 Con: 31.4 ± 3.6	GZFL capsule 2.79 g/day + ECA 2 mg/0.035 mg/day	ECA 2 mg/0.035 mg/day	3 months		Pregnancy, ovulation, LH, Estradiol
Zhao SY 2019 (26)	GZFL:53 Con: 53	GZFL: 28.54 ± 5.02 Con: 27.93 ± 3.21	GZFL pill 12 g/day + clomiphene citrate 100 mg/day	Clomiphene citrate 100 mg/day	4 menstrual cycles	12 months	Pregnancy, ovulation, FSH, TT, Estradiol
Liu W 2022 (28)	GZFL:45 Con: 46	GZFL: 28.76 ± 2.30 Con: 28.14 ± 2.52	GZFL capsule 2.79 g/day + ECA 2 mg/0.035 mg/day + clomiphene citrate 50 g/day × 5	ECA 2 mg/0.035 mg/day + clomiphene citrate 50 g/day × 5	3 menstrual cycles		Pregnancy, ovulation, LH, TT, Estradiol

GZFL, Guizhi Fuling; Con, control; ECA, ethinylestradiol/cyproterone acetate; LH, luteinizing hormone; FSH, follicle-stimulating hormone; TT, total testosterone; HOMA-IR, homeostasis model assessment insulin resistance.

### Ovulation rate

**Figure 2:**
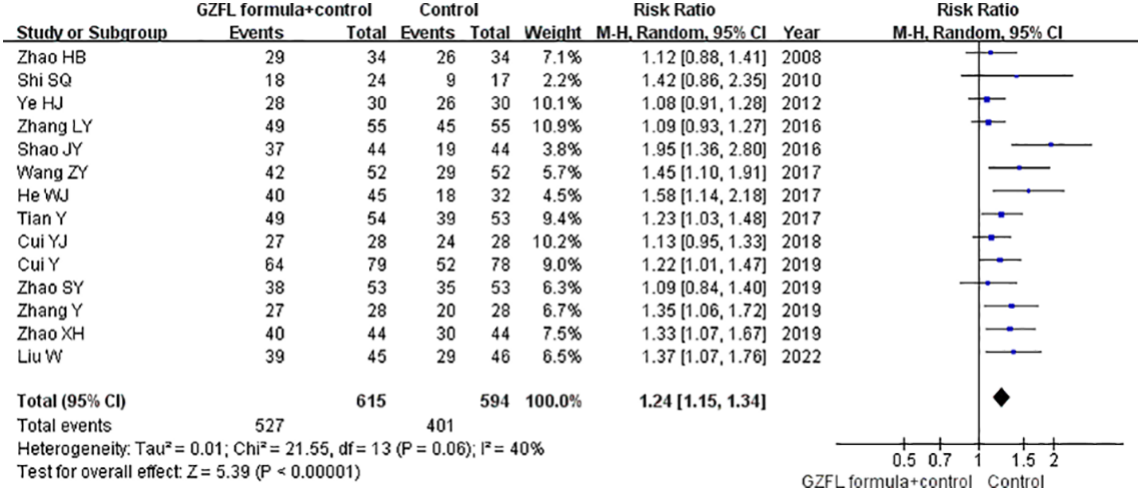
Forest plots showing the pooled ovulation rate comparing the GZFL formula plus Western medicine with the Western medicine alone.

Fourteen trials (13–21, 24–28) reported the effect of the GZFL formula as an adjuvant therapy on the ovulation rate. As shown in **Figure 2**, the GZFL formula plus Western medicine significantly improved the ovulation rate (RR 1.24; 95% CI 1.15–1.34) compared with the Western medicine alone in a random-effect model, with significant heterogeneity (*I^2^* = 39.7%, *p* = 0.063). Leave-out one trial sensitivity analysis showed that the pooled RR of the ovulation rate ranged from 1.21 to 1.26 (all p-values < 0.05). **Table S1** describes the results of subgroup analysis. Both the Begg’s test (*p* = 0.002) and the Egger’s test (*p* = 0.002) suggested the likelihood of publication bias. However, the “trim-and-fill” analysis showed that the corrected pooling RR of ovulation rate was 1.27 (95% CI 1.17–1.38).

### Pregnancy rate

**Figure 3:**
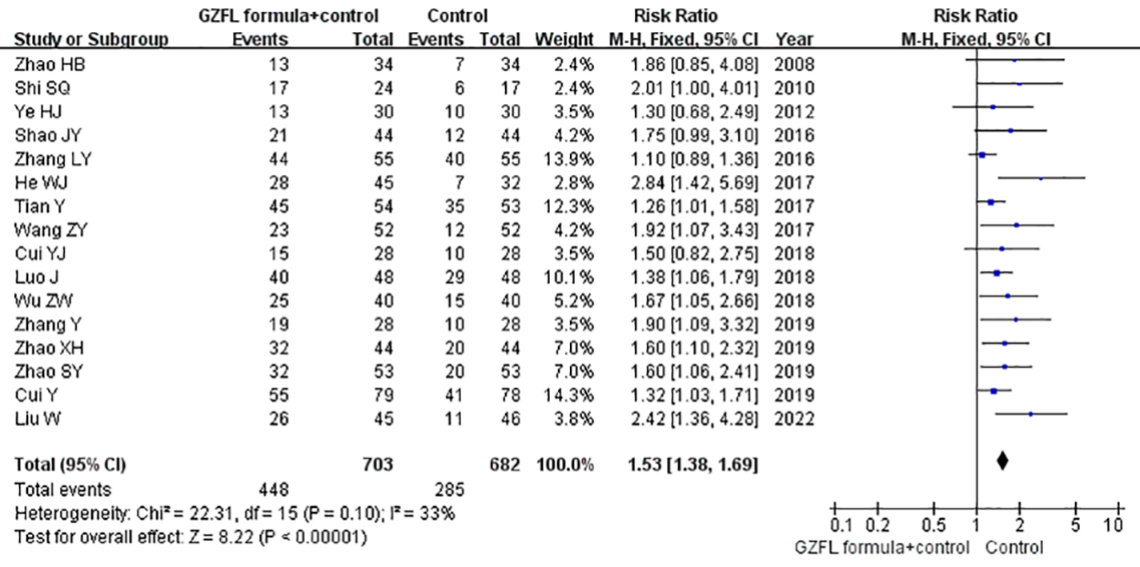
Forest plots showing the pooled pregnancy rate comparing GZFL formula plus Western medicine to the Western medicine alone.

All the included trials reported the effect of the GZFL formula as an adjuvant therapy on the pregnancy rate. As shown in **Figure 3**, the GZFL formula plus Western medicine significantly improved the pregnancy rate (RR 1.53; 95% CI 1.38–1.69) compared with the Western medicine alone, without significant heterogeneity (*I^2^* = 32.8%, *p* = 0.10). Leave-out one trial sensitivity analysis showed that the pooled RR of pregnancy rate ranged from 1.49 to 1.60 (all p-values < 0.05). **Table S2** summarizes the results of subgroup analysis. The Begg’s test (*p* = 0.006) and the Egger’s test (*p* < 0.001) indicated the likelihood of publication bias. However, the “trim-and-fill” analysis suggested that the corrected pooling RR of pregnancy rate was 1.33 (95% CI 1.24–1.41).

### Miscarriage rate

Two trials (17, 22) reported the effect of the GZFL formula as an adjuvant therapy on the miscarriage rate. As shown in **Figure S3**, there was no significant difference on miscarriage rate (RR 0.89; 95% CI 0.36–2.20; *I^2^* = 0.0%, *p* = 0.390) between the GZFL formula plus Western medicine and the Western medicine alone in a fixed-effect model.

### Follicle-stimulating hormone

**Figure 4:**
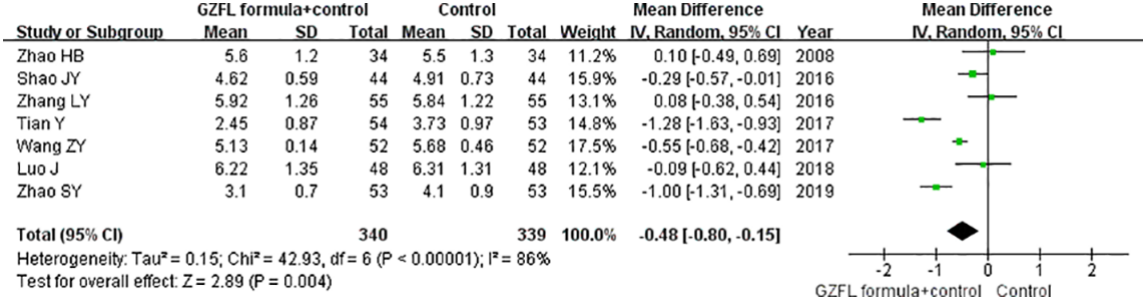
Forest plots showing the pooled serum follicle-stimulating hormone level comparing the GZFL formula plus Western medicine with the Western medicine alone.

Seven trials (13, 16, 17, 19, 20, 22, 26) reported the effect of the GZFL formula on the serum FSH level. As shown in **Figure 4**, a random-effect model meta-analysis indicated that the GZFL formula plus Western medicine significantly reduced the serum FSH level (MD -0.48 U/l; 95% CI -0.80 to -0.15) compared with the Western medicine alone, with significant heterogeneity (*I^2^* = 86.0%, *p* < 0.001). Leave-out one trial sensitivity analysis indicated that the pooled MD of FSH ranged from -0.38 to -0.56 (all p-values < 0.05).

### Luteinizing hormone

**Figure 5:**
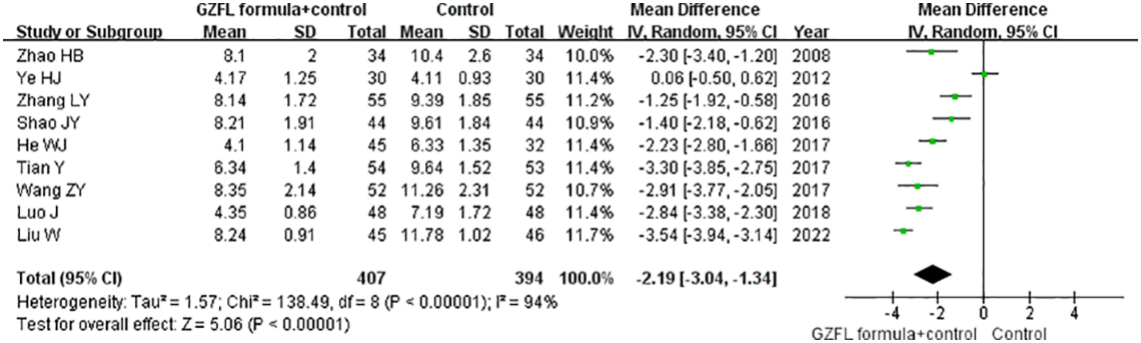
Forest plots showing the pooled serum luteinizing hormone level comparing the GZFL formula plus Western medicine with the Western medicine alone.

Nine trials (13, 15–20, 22, 28) reported the effect of the GZFL formula on the serum LH level. As shown in **Figure 5**, a random-effect model meta-analysis showed that the GZFL formula plus Western medicine significantly reduced the serum LH level (MD -2.19 U/l; 95% CI -3.04 to -1.34) compared with the Western medicine alone, with significant heterogeneity (*I^2^* = 94.0%, *p* < 0.001). Leave-out one trial sensitivity analysis indicated that the pooled MD of LH ranged from -2.02 to -2.50 (all p-values < 0.05).

### Total testosterone

**Figure 6:**
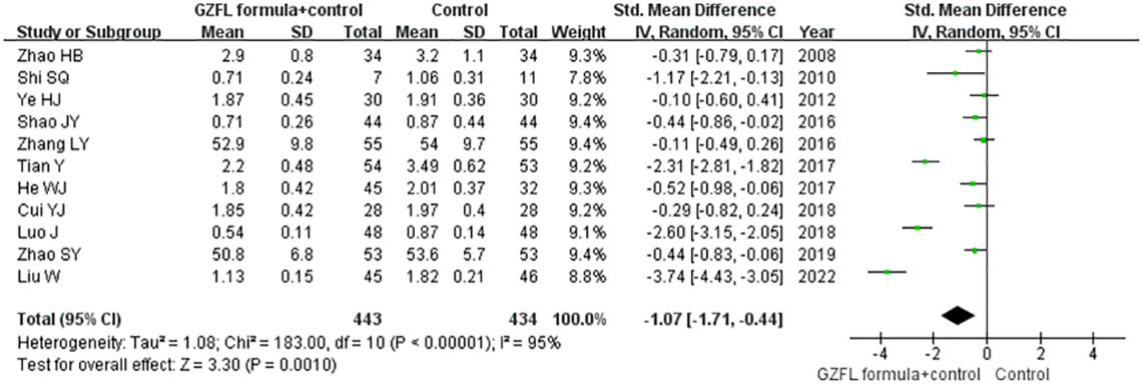
Forest plots showing the pooled serum total testosterone level comparing the GZFL formula plus Western medicine with the Western medicine alone.

Eleven trials (13–19, 21, 22, 26, 28) reported the effect of the GZFL formula on the serum level of total testosterone. As shown in **Figure 6**, a random-effect model meta-analysis suggested that the GZFL formula plus Western medicine significantly reduced the serum total testosterone level (SMD -1.07; 95% CI -1.71 to -0.44) compared with the Western medicine alone, with significant heterogeneity (*I^2^* = 95.0%, *p* < 0.001). Leave-out one trial sensitivity analysis indicated that the pooled SMD of total testosterone ranged from -0.81 to -1.18 (all p-values < 0.05). The Begg’s test (*p* = 0.043) and the Egger’s test (*p* = 0.099) indicated the likelihood of publication bias. However, the “trim-and-fill” analysis suggested that the corrected pooling SMD of serum total testosterone level was unchanged.

### Estradiol

Nine trials (14, 16, 17, 19, 20, 22, 25, 26, 28) reported the effect of the GZFL formula on the serum estradiol level. As shown in **Supplemental Figure S4**, a random-effect model meta-analysis showed that there was no significant difference in serum estradiol level (SMD 0.34; 95% CI -0.25 to 0.94; *I^2^* = 94.0%, *p* < 0.001) between the GZFL formula pus Western medicine and Western medicine groups. Leave-out one trial sensitivity analysis indicated that the pooled SMD of estradiol ranged from 0.11 to 0.45 (all p-values > 0.05).

### Homeostasis model assessment insulin resistance

Three trials (13, 17, 19) reported the effect of the GZFL formula on HOMA-IR level. As shown in **Supplemental Figure S5**, a random-effect model meta-analysis indicated that the GZFL formula plus Western medicine significantly reduced the HOMA-IR level (MD -0.47; 95% CI -0.60 to -0.34) compared with the Western medicine alone, with substantial heterogeneity (*I^2^* = 70.0%, *p* = 0.03). Leave-out one trial sensitivity analysis indicated that the pooled SMD of HOMA-IR ranged from -0.43 to -0.57 (all p-values < 0.05).

### GRADE quality of evidence

The quality of evidence is summarized in **Supplemental Table S3**. The overall certainty of evidence was very low to moderate.

## Discussion

This systematic review and meta-analysis evaluated the add-on effect of the GZFL formula for treating reduced fertility potential in women with PCOS. The main findings of our study were that the GZFL formula in combination with Western medicine significantly improved the ovulation and pregnancy rates in women with PCOS. The GZFL formula as adjuvant therapy could improve approximately 24% and 53% of the ovulation rate and pregnancy rate, respectively, when compared with the Western medicine alone. Moreover, adjuvant treatment with the GZFL formula also significantly reduced the serum FSH, total testosterone, and LH levels as well as HOMA-IR. Considering these findings all together, the GZFL formula as add-on therapy to Western medicine can achieve additional beneficial effects in women with PCOS. It should be noted that adjuvant treatment with the GZFL formula appeared to have no clear effect on the miscarriage rate. However, the certainty of evidence was very low to moderate mainly due to the unclear risk of bias and significant heterogeneity of the included trials.

Our subgroup analysis showed that the add-on effects of the GZFL formula on the ovulation and pregnancy rate were stronger in the studies with more than three menstrual cycles’ treatment. The GZFL capsule appeared to exert better effects on the ovulation and pregnancy rates than the GZFL pill in the subgroup analysis, suggesting that the preparation of GZFL may affect its clinical effect. Regarding the types of Western medicine used, the add-on effect of GZFL appeared to be stronger in the patients who administered ECA or clomiphene citrate alone.

The pathological physiological manifestations of the PCOS are characterized by dysfunction of the hypothalamus–pituitary–ovarian axis and the gonadotropin-releasing hormone secretion, which can result in the secretion of serum FSH, LH, testosterone, and estrogen levels. Our meta-analysis indicated that the GZFL formula combined with Western medicine has more beneficial effects in reducing the serum levels of FSH, LH, and testosterone than Western medicine alone. In addition, the GZFL formula also had additional beneficial effects in reducing insulin resistance. A preclinical study showed that the GZFL formula could ameliorate insulin resistance in PCOS-insulin resistance rat through regulating intestinal flora to control inflammation (29). Moreover, the GZFL formula also inhibited granulosa cell autophagy and promoted follicular development to attenuate ovulation disorder in PCOS-insulin resistance rats (30).

Clomiphene citrate, ECA, metformin, and pioglitazone are used for the treatment of PCOS in the included trials. For patients with ovulatory infertility, clomiphene citrate has long been the gold standard for ovulation induction. Clomiphene citrate remains the first-line pharmacological therapy for infertility associated with PCOS (31). ECA suppresses the male sex hormones (androgens). However, use of ECA could increase the risk for venous thromboembolic complications (32).

Whether the add-on GZFL formula to Western medicine increases the adverse events is a big concern. Only one trial (27) reported adverse events including rash, headache, and insomnia. There was no significant difference in adverse events between the GZFL formula and control group. Adding the GZFL formula to Western medicine appeared to not increase the adverse events in this trial. However, we were unable to draw a firm conclusion about the safety of the GZFL formula combined with Western medicine. Future RCTs are warranted to investigate whether the GZFL formula as adjuvant therapy to Western medicine increases the adverse events.

The current systematic review and meta-analysis had important clinical implications. Adding the GZFL formula to Western medicine could significantly improve the ovulation and pregnancy rates. Regarding the preparation of the GZFL formula, the effect of the GZFL capsule on the ovulation and pregnancy rates appeared to be stronger than the GZFL pill. More than three menstrual cycles’ treatment could exert better effects than that with less than three menstrual cycles. In addition, the add-on effect of the GZFL formula was more pronounced in combination with ECA or clomiphene citrate alone. Based on the theory of TCM, the GZFL formula is more suitable for patients with Qi stagnation and blood stasis syndrome. However, TCM syndrome differentiation was not considered in the majority of included trials. Future trials should consider the TCM syndrome differentiation in the process of patient selection.

Our systematic review and meta-analysis had certain limitations. First, a major concern is the methodological flaws of the analyzed trials. Only six trials clearly report the method of randomization. Nevertheless, all the included trials did not mention the allocation concealment and blind method. Second, majority of the included trials did not take into account the TCM syndrome differentiation in their diagnostic procedures, which could have resulted in potential selection bias of patients. Third, there was significant heterogeneity in the pooling serum hormone level and HOMA-IR. Different patients’ characteristics, course of treatment, types of GZFL formula, and regimens of Western medicine may contribute to the observed heterogeneity. Fourth, all included original RCTs were written in Chinese, which gives difficulty for the readers to evaluate the quality of original trials. Finally, results of stratified analysis were potentially unreliable because of the small number of trials included in the subgroups.

## Conclusions

The GZFL formula as adjuvant therapy to Western medicine can improve the ovulation and pregnancy rate in women with PCOS. The beneficial effects of the GZFL formula may correlate with reducing serum FSH, total testosterone, LH, and ameliorating insulin resistance. However, more well-designed RCTs with larger samples and multicenter trials are required to confirm the current findings due to the uncertainty of evidence.

## Data availability statement

The original contributions presented in the study are included in the article/**Supplementary Material**. Further inquiries can be directed to the corresponding author.

## Author contributions

Study conception/design and interpretation of data: WM; literature search, data extraction, quality assessment, and statistical analysis: AR and NT; writing of the manuscript: LE; revising of the manuscript: WM. All authors read and approved the final version of manuscript.

## References

[1] Deswal R Narwal V Dang A Pundir CS . The prevalence of polycystic ovary syndrome: A brief systematic review. J Hum Reprod Sci (2020) 13(4):261–71. doi: 10.4103/jhrs.JHRS_95_18 PMC7879843 33627974

[2] Helvaci N Yildiz BO . Polycystic ovary syndrome and aging: Health implications after menopause. Maturitas (2020) 139:12–9. doi: 10.1016/j.maturitas.2020.05.013 32747035

[3] Palomba S . Is fertility reduced in ovulatory women with polycystic ovary syndrome? an opinion paper. Hum Reprod (2021) 36(9):2421–8. doi: 10.1093/humrep/deab181 34333641

[4] Palomba S Daolio J La Sala GB . Oocyte competence in women with polycystic ovary syndrome. Trends Endocrinol Metab (2017) 28(3):186–98. doi: 10.1016/j.tem.2016.11.008 27988256

[5] Cunha A Póvoa AM . Infertility management in women with polycystic ovary syndrome: A review. Porto BioMed J (2021) 6(1):e116. doi: 10.1097/j.pbj.0000000000000116 33532657 PMC7846416

[6] Zehravi M Maqbool M Ara I . Polycystic ovary syndrome and infertility: An update. Int J Adolesc Med Health (2021) 34(2):1–9. doi: 10.1515/ijamh-2021-0073 34293835

[7] Qin X . Application and mechanism of guizhi fuling pill for management of gynecological diseases. J Guangxi Univ Chin Med (2021) 24(1):61–4.

[8] Ong M Peng J Jin X Qu X . Chinese Herbal medicine for the optimal management of polycystic ovary syndrome. Am J Chin Med (2017) 45(3):405–22. doi: 10.1142/S0192415X17500252 28359195

[9] Zhang YY Xu ZY Zhou LS Wang CY Yu J Ding CF . Meta analysis of guizhi fuling pill as an adjuvant therapy for treatment of polycystic ovary syndrome. Zhejiang J Integrated Traditional Chin Western Med (2021) 31(6):572–8.

[10] Liberati A Altman DG Tetzlaff J Mulrow C Gotzsche PC Ioannidis JP . The PRISMA statement for reporting systematic reviews and meta-analyses of studies that evaluate health care interventions: Explanation and elaboration. J Clin Epidemiol (2009) 62(10):e1–34. doi: 10.1016/j.jclinepi.2009.06.006S0895-4356(09)00180-2 19631507

[11] Begg CB Mazumdar M . Operating characteristics of a rank correlation test for publication bias. Biometrics (1994) 50(4):1088–101. doi: 10.2307/2533446 7786990

[12] Egger M Davey Smith G Schneider M Minder C . Bias in meta-analysis detected by a simple, graphical test. BMJ (1997) 315(7109):629–34. doi: 10.1136/bmj.315.7109.629 PMC2127453 9310563

[13] Zhao HB He YR . Clinical observation of effect of clomiphene citrate combined with cassia tuckahoe capsule on infertile polycystic ovary syndrome patients. China Trop Med (2008) 8(11):1942–3.

[14] Shi SQ Huang BZ Wang JY Wang F . The efficacy of guizhifuling capsule promoting ovulation in non-obese polycystic ovary syndrome patients. China Med (2010) 5(12):1176–7. doi: 10.3760/cma.j,issn.1673-4777.2010.12.029

[15] Ye HJ Jiang YJ Li AP Yu Y . Clinical observation on treating polycystic ovary syndrome with stertility with guizhi fuling capsule and Diane-35 and clomifene citrate. Chin J Clin Pharmacol Ther (2012) 17(6):691–5.

[16] Shao JY Liu J . Influence of guizhi fuling capsule combined with clomiphene on the hormone level and pregnancy rate of patients with polycystic ovarian syndrome. Henan Traditional Chin Med (2016) 36(5):899–901. doi: 10.16367/j.issn.1003-5028.2016.05.0379

[17] Zhang LY Yin WQ . Effect of guizhi fuling pill treatment for polycystic ovary syndrome patients with insulin resistance. J Chin Medicinal Materials (2016) 39(7):1161–3. doi: 10.13863/j.issn1001-4454.2016.07.051

[18] He WJ Zhao R . Efficacy of cinnamon twig and poria bolus combined with diane-35 in the treatment of infertility induced by polycystic ovary syndrome and their influence on serum visfatin and high-sensitivity c-reactive protein. Guangxi Med J (2017) 39(2):165–8. doi: 10.11675/j.issn.0253-4304.2017.02.08

[19] Tian Y Gao XL . Guizhi fuling gum combined with western medicine in the treatment of polycystic ovary syndrome and its effects on endocrine metabolism and ovulation. Shanxi Tradiitional Chin Med (2017) 38(4):444–5. doi: 10.3969/j.issn.1000-7369.2017.04.017

[20] Wang ZY . Effect of ethinylestradiol and cyproterone combined with guizhi fuling capsule on infertility of polycystic ovary syndrome, ovulation rate and pregnancy outcome. Pract Clin J Integrated Traditional Chin Western Med (2017) 17(6):77–8. doi: 10.13638/j.issn.1671-4040.2017.06.050

[21] Cui YJ . Clinical efficacy of guizhi fuling combined with Diane-35 in the treatment of infertility with polycystic ovary syndrome. J Imaging Res Med Appl (2018) 2(10):215–6.

[22] Luo J Nie RQ Lin LY . Influence of taie-35 and clomifen combined with laurel cocos poria pill on the pregnancy and psychological state of polycystic ovary syndrome patients. Drug Eval Res (2018) 41(7):1300–3. doi: 10.7501/j.issn.1674-6376.2018.07.028

[23] Wu ZW . Clinical efficacy of guizhi fuling combined with Diane-35 in the treatment of infertility with polycystic ovary syndrome. Diet Health (2018) 5(14):87.

[24] Zhang Y . Guizhi fuling capsule combined with ethinylestradiol, cyproterone and metformin in the treatment of 56 cases of polycystic ovary syndrome. J North Pharm (2019) 16(1):136–7.

[25] Zhao XH . Clinical observation of guizhi fuling capsule combined with ethinylestradiol and cyproterone in the treatment of polycystic ovary syndrome. Capital Med (2019) 26(19):65.

[26] Zhao SY Wei YY . Effect of guizhi fuling capsule on ovarian function and clinical symptoms in patients with polycystic ovary syndrome complicated with insulin resistance. Electronic J Of Pract Gynecologic Endocrinol (2019) 6(23):106–7.

[27] Cui Y Li SP Li Y Yin XY . Effect of guizhi fuling pill combined with pioglitazone on serum RANTES, MCP-1 and pregnancy rate in patients with polycystic ovary syndrome. World J Complex Med (2019) 5(5):81–3.

[28] Liu W Du XH . Effects of guizhi fuling capsule combined with ethinylestradiol and cyproterone pretreatment on endocrine hormone level and ovulation induction in infertile patients with polycystic ovary syndrome. Contemp Med (2022) 28(7):83–5. doi: 10.3969/j.issn.1009-4393.2022.07.028

[29] Zhu Y Li Y Liu M Hu XD Zhu HQ . Guizhi fuling wan, Chinese herbal medicine, ameliorates insulin sensitivity in PCOS model rats with insulin resistance *via* remodeling intestinal homeostasis. Front Endocrinol (Lausanne) (2020) 11:575. doi: 10.3389/fendo.2020.00575 32973686 PMC7482315

[30] Liu M Zhu H Zhu Y Hu X . Guizhi fuling wan reduces autophagy of granulosa cell in rats with polycystic ovary syndrome *via* restoring the PI3K/AKT/mTOR signaling pathway. J Ethnopharmacol (2021) 270:113821. doi: 10.1016/j.jep.2021.113821 33460753

[31] Costello MF Misso ML Balen A Boyle J Devoto L Garad RM . A brief update on the evidence supporting the treatment of infertility in polycystic ovary syndrome. Aust N Z J Obstet Gynaecol (2019) 59(6):867–73. doi: 10.1111/ajo.13051 31514246

[32] Ruan X Kubba A Aguilar A Mueck AO . Use of cyproterone acetate/ethinylestradiol in polycystic ovary syndrome: Rationale and practical aspects. Eur J Contracept Reprod Health Care (2017) 22(3):183–90. doi: 10.1080/13625187.2017.1317735 28463030

