# Supplementary material for: Add-on effect of the Guizhi Fuling formula for management of reduced fertility potential in women with polycystic ovary syndrome: A systematic review and meta-analysis of randomized controlled trials
Source: Front Endocrinol (Lausanne). 2023 Apr 18;13:995106. doi: 10.3389/fendo.2022.995106 (PMC10153095; doi:10.3389/fendo.2022.995106)
Supplement: Supplementary file 1 [file Table_1.doc]

~~Table S1 Results of subgroup analysis on ovulation rate~~

| Subgroups | Number of studies | Pooled RR | 95% CI | Heterogeneity between studies |
| --- | --- | --- | --- | --- |
| Publication year  ≥ 2018  <2018 | 6  8 | 1.23  1.28 | 1.12 to 1.34  1.11 to 1.47 | *p*=0.567; I^2^ =0.0%  *p*=0.008; I^2^ =63.0% |
| Form of GZFL formula  Capsule  Pill | 9  4 | 1.30  1.19 | 1.16 to 1.45  1.03 to 1.36 | *p*=0.083; I^2^ =42.6%  *p*=0.181; I^2^ =38.6% |
| Course of treatment  ≥3 months  < 3 months | 5  9 | 1.39  1.19 | 1.16 to 1.66  1.10 to 1.28 | *p*=0.116; I^2^ =46.2%  *p*=0.294; I^2^ =16.7% |
| Type of Western medicine  ECA  ECA+ Metformin  Clomiphene citrate | 4  3  4 | 1.32  1.19  1.31 | 1.11 to 1.57  1.06 to 1.34  1.00 to 1.72 | *p*=0.101; I^2^ =51.8%  *p*=0.296; I^2^ =18.0%  *p*=0035; I^2^ =65.2% |

GZFL, Guizhi Fuling; RR, risk ratio; CI, confidence interval; ECA, ethinylestradiol and cyproterone acetate tablet
