# Supplementary material for: Add-on effect of the Guizhi Fuling formula for management of reduced fertility potential in women with polycystic ovary syndrome: A systematic review and meta-analysis of randomized controlled trials
Source: Front Endocrinol (Lausanne). 2023 Apr 18;13:995106. doi: 10.3389/fendo.2022.995106 (PMC10153095; doi:10.3389/fendo.2022.995106)
Supplement: Supplementary file 2 [file Table_2.doc]

~~Table S2 Results of subgroup analysis on pregnancy rate~~

| Subgroups | Number of studies | Pooled RR | 95% CI | Heterogeneity between studies |
| --- | --- | --- | --- | --- |
| Publication year  ≥ 2018  <2018 | 8  8 | 1.56  1.49 | 1.36 to 1.79  1.28 to 1.73 | *p*=0.625; I^2^ =0.0%  *p*=0.028; I^2^ =55.4% |
| Form of GZFL formula  Capsule  Pill | 10  5 | 1.66  1.40 | 1.43 to 1.93  1.22 to 1.61 | *p*=0.468; I^2^ =0.0%  *p*=0.045; I^2^ =59.0% |
| Course of treatment  ≥3 months  < 3 months | 6  10 | 1.75  1.45 | 1.41 to 2.18  1.30 to 1.63 | *p*=0.991; I^2^ =0.0%  *p*=0.052; I^2^ =46.4% |
| Type of Western medicine  ECA  ECA+ Metformin  Clomiphene citrate | 6  3  4 | 1.68  1.26  1.74 | 1.40 to 2.01  1.08 to 1.47  1.31 to 2.21 | *p*=0.440; I^2^ =0.0%  *p*=0.157; I^2^ =46.1%  *p*=0.950; I^2^ =0.0% |

GZFL, Guizhi Fuling; RR, risk ratio; CI, confidence interval; ECA, ethinylestradiol and cyproterone acetate tablet
