# Supplementary material for: Add-on effect of the Guizhi Fuling formula for management of reduced fertility potential in women with polycystic ovary syndrome: A systematic review and meta-analysis of randomized controlled trials
Source: Front Endocrinol (Lausanne). 2023 Apr 18;13:995106. doi: 10.3389/fendo.2022.995106 (PMC10153095; doi:10.3389/fendo.2022.995106)
Supplement: Supplementary file 3 [file Table_3.docx]

Table S3 GRADE of evidence of outcomes of the included trials

| Outcomes |  | Certainty assessment | | | | | Effect | | Certainty | Importance |
| --- | --- | --- | --- | --- | --- | --- | --- | --- | --- | --- |
|  | No. of trials | Risk of bias | Inconsistency | Indirectness | Imprecision | Publication bias | No. of patients | Effect sizes |  |  |
| Ovulation rate | 14 | Serious | Serious | Not serious | Not serious | Likelihood# | 1209 | RR 1.24 (95%CI 1.15 to 1.34) | ⊕⊕⊝⊝ **Low** | Critical |
| Pregnancy rate | 16 | Serious | Not serious | Not serious | Not serious | Likelihood# | 1385 | RR 1.53 (95%CI 1.38 to 1.69) | ⊕⊕⊕⊝ **Moderate** | Critical |
| Miscarriage rate | 2 | Serious | Not serious | Serious | Serious | Not detected^ | 650 | RR 0.86 (95%CI 0.39 to 2.20) | ⊕⊝⊝⊝ **Very low** | Critical |
| Follicle-stimulating hormone | 7 | Serious | Serious | Not serious | Not serious | Not detected^ | 679 | MD -0.48 U/L (95%CI -0.80 to -0.15) | ⊕⊝⊝⊝ **Very low** | Important |
| Luteinizing hormone | 9 | Serious | Serious | Not serious | Not serious | Not detected^ | 801 | MD -2.19 U/L (95%CI -3.04 to -1.34) | ⊕⊝⊝⊝ **Very low** | Important |
| Total testosterone | 11 | Serious | Serious | Not serious | Not serious | Likelihood# | 877 | SMD -1.07 (95%CI -1.71 to -0.44) | ⊕⊕⊝⊝ **Low** | Important |
| Estradiol | 9 | Serious | Serious | Not serious | Not serious | Not detected^ | 808 | SMD 0.34 (95%CI -0.25 to 0.94) | ⊕⊝⊝⊝ **Very low** | Important |
| HOMA-IR | 3 | Serious | Serious | Not serious | Not serious | Not detected^ | 285 | MD-0.47 (95% CI -0.60 to -0.34) | ⊕⊝⊝⊝ **Very low** | Important |

RR, risk ratios; MD, mean difference; SMD, standardized mean difference CI, confidence interval; HOMA-IR, homeostasis model assessment insulin resistance. Risk of bias; serious, study with unclear risk of bias; Inconsistency: Serious, I ^2^ >50%. Indirectness of evidence, no indirectness of evidence was found in any study. Imprecision (based on sample size): Serious, n < 500 participants. # Begg’s and Egger’s test suggested the presence of publication bias and trim-and-fill analysis did alter the originally statistical significance. ^Not detected due to the number of trials less than the recommended arbitrary minimum number of 10.

GRADE Working Group grades of evidence
**High quality:** Further research is very unlikely to change our confidence in the estimate of effect.
**Moderate quality:** Further research is likely to have an important impact on our confidence in the estimate of effect and may change the estimate.
**Low quality:** Further research is very likely to have an important impact on our confidence in the estimate of effect and is likely to change the estimate.
**Very low quality:** We are very uncertain about the estimate.
