# Supplementary material for: Add-on effect of the Guizhi Fuling formula for management of reduced fertility potential in women with polycystic ovary syndrome: A systematic review and meta-analysis of randomized controlled trials
Source: Front Endocrinol (Lausanne). 2023 Apr 18;13:995106. doi: 10.3389/fendo.2022.995106 (PMC10153095; doi:10.3389/fendo.2022.995106)

Supplemental Figure S1. Risk of bias graph


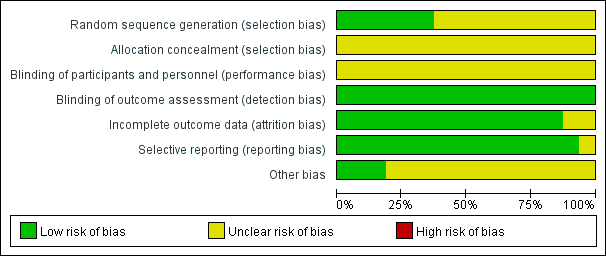


Supplemental Figure S2. Risk of bias summary


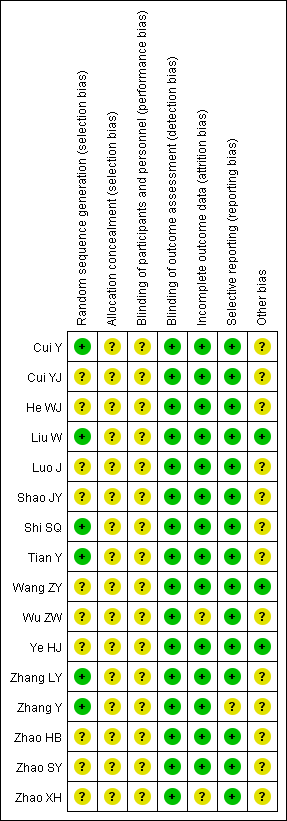


Supplemental Figure S3. Forest plots showing the pooled miscarriage rate comparing GZFL formula plus Western medicine to the Western medicine alone.

Supplemental Figure S4. Forest plots showing the pooling serum estradiol level comparing GZFL formula plus Western medicine to the Western medicine alone.


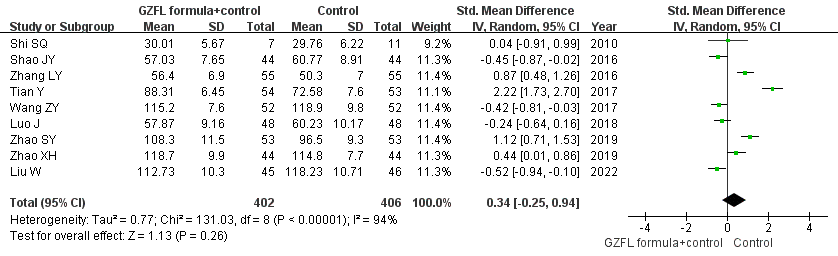


Supplemental Figure S5. Forest plots showing the pooling homeostasis model assessment insulin resistance comparing GZFL formula plus Western medicine to the Western medicine alone.


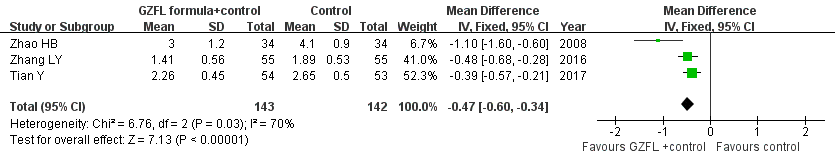

Supplement: Supplementary file 4 [file Table_4.docx]
