# Supplementary material for: Add-on effect of the Guizhi Fuling formula for management of reduced fertility potential in women with polycystic ovary syndrome: A systematic review and meta-analysis of randomized controlled trials
Source: Front Endocrinol (Lausanne). 2023 Apr 18;13:995106. doi: 10.3389/fendo.2022.995106 (PMC10153095; doi:10.3389/fendo.2022.995106)
Supplement: Supplementary file 5 [file Table_5.doc]

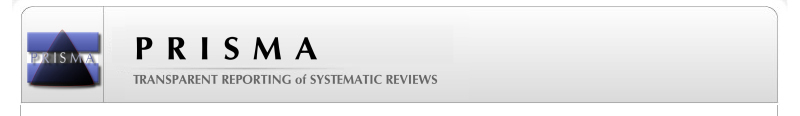
**PRISMA 2009 Flow Diagram**

**Screening**

**Included**

**Eligibility**

**Identification**

Records identified through database searching
(n =230 )

Additional records identified through other sources
(n = 0 )

Records after duplicates removed
(n =138)

Records screened
(n =138)

Records excluded
(n =93)

Full-text articles assessed for eligibility
(n =45)

29 trials excluded with reasons: No randomized controlled trial (n = 3) Modified Guizhi Fuling formula as intervention (n = 7); Combined with complementary and alternative therapy as intervention (n = 5); Not use Guizhi Fuling formula as adjunctive therapy (n = 1); Outcome measures were not of interest (n = 9); Suspected plagiarism or duplicate publication (n = 3); study protocol (n = 1)

Articles included in qualitative synthesis
(n =16 )

16 studies included in the meta-analysis
